# Effects of contrasting herbivore cues on seedlings of a long-lived woody tree: growth, chemistry, and resistance to herbivores

**DOI:** 10.1093/aobpla/plag026

**Published:** 2026-06-10

**Authors:** Santhi P Bhavanam, Evan L Preisser, Colin M Orians

**Affiliations:** Department of Biology, Tufts University, Medford, MA 02155, United States; Department of Biological Sciences, University of Rhode Island, Kingston, RI 02881, United States; Department of Biology, Tufts University, Medford, MA 02155, United States

**Keywords:** dusky slug, hardwood species, induction, secondary metabolites, resistance

## Abstract

Plant-induced defences against herbivores have the potential to modify plant growth, fitness, and the outcome of plant–herbivore interactions. Plants use a variety of cues to initiate the induction of defences, including physical tissue damage, cues released by damaged plant tissues of neighbouring plants (e.g. methyl jasmonate, MeJA), and cues that indicate herbivore presence but are not associated with herbivore damage (e.g. locomotion mucus of herbivorous molluscs). Evidence shows that both herbivore-damage and herbivore-presence cues can generate induced defences in herbaceous plants over relatively short time periods. However, the immediate and long-term effects of these cues on seedlings of deciduous broad-leaved woody plants remain poorly understood. Here, we compare the effects of two cues, MeJA and locomotion mucus of the dusky slug *Arion subfuscus*, on the growth and defence traits of sugar maple, *Acer saccharum*, seedlings at multiple timepoints. Specifically, we exposed 4-week-old seedlings to these cues in a greenhouse experiment and quantified their effects on seedling growth, total phenolics, and growth of spongy moth *Lymantria dispar* across the seedlings’ growing season. As expected, MeJA had a negative effect on seedling growth, a positive effect on total phenolics, and no effect on the growth of *L. dispar*. In contrast, the slug locomotion mucus did not negatively affect growth or change total phenolic levels. Our results highlight that herbivore cues can induce responses in seedling growth and defence in deciduous broad-leaved woody plant seedlings; however, these responses vary with cue type and the induced responses decay for some seedling traits but not others.

## Teaser

Induced responses to herbivory cues are well-documented in herbaceous plant species but are less studied in seedlings of deciduous broad-leaved woody plants. Using *Acer saccharum*, we show that exposing 4-week-old seedlings to methyl jasmonate (MeJA) reduced seedling growth and increased total phenolic concentration, though these induced responses decayed over time. Conversely, slug mucus treatment did not alter seedling growth or total phenolics. Further, neither MeJA nor slug mucus treatment affected the growth of spongy moth larvae. Our study demonstrates variable induced responses to different herbivory cues in these seedlings.

## Introduction

Plants have responded to the threat of herbivory by evolving defences such as damage-induced changes in growth and defences (Karban *et al.* 1997, Karban and Baldwin 1997, Karban and Myers 1989, Kessler and Mueller 2024, Nykänen and Koricheva 2004, Van Loon 2000, Walters *et al.* 2007) that may interfere with herbivore preference and performance (Agrawal 1999, Karban *et al.* 1997, Karban and Myers 1989, Kessler and Mueller 2024, Nykänen and Koricheva 2004). A broad array of cues has been found to trigger-induced defences in plants, including physical tissue damage and cues emitted by damaged plant tissues from neighbouring plants (Karban and Baldwin 1997). In addition to cues associated with actual plant damage, plants may also induce defence in response to cues of herbivore presence, presumably because such cues indicate the potential for future herbivory. For example, oviposition by herbivores and trichome stimulation by moving herbivores may trigger defence induction (Hilker and Fatouros 2016, Tian *et al.* 2012). Recent evidence also indicates that chemicals produced by herbivores that are not directly related to herbivore feeding will also lead to the induction of defence. For instance, sex pheromones (Helms *et al.* 2013, 2017) and mucus produced by molluscs for locomotion (Falk *et al.* 2014, Orrock 2013, Orrock *et al.* 2018, Pellegrini *et al.* 2024) lead to changes in plant growth and defence.

While well-documented in herbaceous plants, the effect of contrasting herbivore cues is much less studied in woody plants (Calf *et al.* 2019, Desurmont *et al.* 2016). For example, Mann *et al.* (2021) reported that volatile emissions were higher in plants infested with *Spodoptera littoralis* compared to those infested by *Arion* spp. in different plant species. MeJA, a volatile compound, can induce changes in growth, defences, and resistance in both herbaceous and woody plants (Berggren *et al.* 2023, Chen *et al.* 2021, Huynh *et al.* 2024, Krokene *et al.* 2023, Moreira *et al.* 2012, Puentes *et al.* 2021, Rowe *et al.* 2012, Scott *et al.* 2019, Tianzi *et al.* 2018), but the effects of slug locomotion mucus remain unknown in woody plants. Furthermore, induced responses are expected to be especially important in young seedlings, when resources are relatively limited, and the threat of herbivory is real but variable (Barton and Koricheva 2010). Induction in woody plants at the seedling stage may be strongest because they often contain relatively low concentrations of constitutive defence compounds (Boege and Marquis 2005). In this study, we test whether MeJA, a volatile compound known to induce changes in undamaged plants, and gastropod locomotion mucus alter growth and defences in seedlings of a deciduous broad-leaved woody plant species.

Gastropods (slugs and snails) are major consumers of seedlings in temperate regions (Albrectsen *et al.* 2007, Hanley *et al.* 1995), so understanding induced responses to herbivore damage and non-damage cues is likely to be very important in deciduous woody plant species. Previous work has shown that slug feeding upregulates several phytohormone pathways, including jasmonic acid (JA) pathway in *Solanum dulcamara* (Calf *et al.* 2020) and salicylic acid (SA) pathway in *Arabidopsis thaliana* (Meldau *et al.* 2014). In addition, the locomotion mucus of gastropods can increase levels of JA in *A. thaliana* (Falk *et al.* 2014), antioxidant and antidefense enzymes in *Solanum lycopersicum* (Orrock *et al.* 2018), and ultimately increase resistance to herbivory (Orrock 2013). Recently, Pellegrini *et al.* (2024) showed that seeds and seedlings of *Brassica nigra* exposed to slug mucus subsequently experienced 88% less damage by slugs. However, whether similar responses exist in deciduous broad-leaved woody plants is still unknown.

Slugs also can cause extensive mortality of woody plant seedlings in temperate ecosystems (Albrectsen *et al.* 2007, Gardescu 2003, Pigot and Leather 2008), but patterns of herbivory vary with seedling age. In general, young seedlings are more susceptible to slugs than older ones (Albrectsen *et al.* 2004, Elger *et al.* 2009, Orians *et al.* 2010) as plant defences, including lignification, change with plant age. Such shifts in resistance are expected when young seedlings do not have sufficient root mass, storage tissues, or resources for both defence and growth (Barton and Boege 2017, Boege *et al.* 2011, Boege and Marquis 2005, Dylewski *et al.* 2024). In this context, a trade-off between growth and defence is expected. However, trade-offs in older plants may not be as common as in young plants since older plants have more resources to allocate for growth and defences (see Albrectsen *et al.* 2004, Cope *et al.* 2021, Ni *et al.* 2020, Orians *et al.* 2010). Yet, if plant susceptibility to an herbivore decreases rapidly with ontogeny/size, then induced increases in growth may shorten the window of vulnerability and lead plants to prioritize growth over defence (Boege and Marquis 2005, Hódar *et al.* 2008).

To test whether contrasting cues, MeJA and slug locomotion mucus, differentially alter the relationships among growth, defence, and herbivore performance, we studied the responses of sugar maple, *Acer saccharum*, seedlings over the growing season (3–88 days post final cue application). Induced responses tend to be ephemeral, with peak induction occurring immediately after herbivore cue treatment, followed by a decline in plants (Karban and Baldwin 1997). For example, Stevens and Lindroth (2005) showed that the difference in condensed tannins between damaged and undamaged plants was pronounced 1 week after damage but absent 8 weeks later. Thus, we expected to find a strong initial induced response in treated seedlings that would decay (become more like control seedlings) with increasing time post-cue application.

Our preliminary feeding trial showed that slugs did not or only minimally (<1%) consumed sugar maple leaves, at 16 h from the initiation of the feeding trial, while slugs consumed lettuce leaves completely (in less than 2 h). No measurements of leaf area consumed by slugs were taken, as the results were obvious. Given that slugs avoid mature sugar maple leaves, we explored how cue-induced changes altered the performance of a leaf-chewing herbivore, *Lymantria dispar* L. Because both chewing caterpillars and slugs can upregulate the same JA defence pathway, we would expect that prior exposure to these cues would also alter the performance of leaf-chewing herbivores. More specifically, we explored whether MeJA and slug locomotion mucus cues (1) alter the growth and total phenolics of *A. saccharum* seedlings, (2) induce resistance in seedlings to *L. dispar*, and (3) induced responses decay over time. We hypothesized that both MeJA and slug locomotion mucus would slow growth, increase total phenolics, and decrease seedling susceptibility to *L. dispar*, and that these effects would be transient and dissipate over time.

## Materials and methods

### Study system

#### Arion subfuscus

The dusky slug, *Arion subfuscus*, was introduced into North America in 1800s and is now well-established in Northeastern forests of the USA and Canada (Chichester and Getz 1969). Unlike native slugs that feed on fungi, *A. subfuscus* is common in the forest understory where they feed on woody plants, including maple seedlings, and this often results in high mortality (Gardescu 2003, Nystrand and Granstrom 1997). While slugs readily consume seeds and cotyledons, they avoid consuming older seedlings and mature leaves (Bhavanam, S., Headrick, K., and Pellegrini, B., personal observation).

#### Acer saccharum

Sugar maples are foundational trees in eastern North America (Cleavitt *et al.* 2021, Copenheaver *et al.* 2020). They are highly shade tolerant, and seedlings are common in the forest understory (Bognounou *et al.* 2023, Copenheaver *et al.* 2020, Parsons *et al.* 2025). In preliminary assays, we found that *A. subfuscus* avoids eating leaf tissue of older maple seedlings. Therefore, we focused on the effects of both MeJA and slug locomotion mucus cues on the performance of *L. dispar*, a leaf-chewing herbivore that feeds on *A. saccharum* (Barbehenn *et al.* 2009, 2013) throughout the invaded range and can cause major damage to even mature trees during outbreaks (Gardescu 2003).

#### Sugar maple planting

Sugar maple samaras were sourced from Sheffield’s Seed Co., NY, USA (Lot # 1829258, PA, USA). For this study, we used samaras ranging between 90 and 160 mg, and all samaras heavier than 90 mg had a seed. To initiate seed germination, samaras were soaked in tap water for 24 h at room temperature, then washed for three times in tap water and cold stratified on water-saturated paper towels in Ziploc bags at 4°C in a refrigerator until germination. Samaras were surface-sterilized once with 10% bleach solution during cold stratification. Once seeds started germinating (radicle emergence began at ∼60 days), samaras were checked for germination every third day, and those that germinated within few days of each other were grouped and planted on the same day in a greenhouse at Tufts University, Medford, MA, USA. A single samara was placed at the centre of a 2.5″ diameter × 10″ long plastic pots (Deepot Cells & Trays, Stuewe and Sons Inc, OR, USA) that was filled with a soil mix comprised of loamy coarse sand soil (Read Custom Soils, MA, USA), peat moss, and sand in 1:2:2 ratio. Each pot was stacked in a 12″L × 14.8″W × 9.4″H support tray (Stuewe and Sons Inc, OR, USA). One week after seedling emergence, seedlings were fertilized weekly with 15 ml of 2 g/L 20–20–20 N–P–K Scotts water soluble fertilizer solution that also contained full strength of Ca (calcium nitrate), and Mg (magnesium sulphate) Hoagland solution (Hoagland and Arnon 1950). At 3 weeks after seedling emergence, seedlings were provided weekly with 15 ml of a full-strength Hoagland micronutrient solution (Hoagland and Arnon 1950). Seedlings were watered as needed and left to grow under natural light conditions in the greenhouse from early summer until early fall.

Treatments began at 4 weeks post-emergence. At this stage, seedlings had one pair of fully expanded leaves, and new pairs of leaves were starting to develop under the greenhouse conditions. Sixty 4-week-old seedlings were equally divided and randomly assigned to one of the three cue type treatments: (1) control; (2) 5 mM MeJA; and (3) locomotion mucus of *A. subfuscus* (hereafter ‘slug mucus’). Our preliminary study with different concentrations of MeJA (5 and 10 mM) showed that a concentration of 5 mM MeJA does not cause visible leaf damage in sugar maple seedlings. Initial seedling height, from cotyledon scar to the shoot tip (hereafter ‘initial height’), was taken the day before the first treatment application.

### Herbivore cue preparation and application

A 5 mM MeJA solution was prepared by dissolving 224 µl of MeJA in 500 µl of absolute ethanol. Then, the aliquot was added to 200 ml deionized water containing 250 µl of the carrier, Triton-X 100 (0.125% v/v). A solution containing carrier and ethanol without MeJA was prepared for the control and slug mucus treatments.

To prepare the slug mucus cue, several *A. subfuscus* were collected from woody areas in Burlington, MA, USA during the spring and summer of 2023. Collected slugs were transported in plastic containers to Tufts University, Medford, MA, USA where they were reared in 40 L terrariums that were half-filled with soil, leaf litter, and bark under lab conditions. The top of the terrariums was covered with a fine mesh and closed with a lid. Each terrarium housed ∼50 slugs. Every third day, the terrarium soil was moistened with deionized water, and slugs were provided with store-bought organic iceberg lettuce. Unconsumed lettuce was removed periodically.

To collect the locomotion mucus from slugs, 2 days before the cue application, 20 slugs (slug mass range: 1.5–3 g) from a single terrarium were removed and starved in separate 90 mm Petri dishes lined with moistened filter paper for 6 h to maximize frass removal. For each slug mucus cue preparation date, slugs from a different terrarium were used, and each slug was used only once for mucus collection. Each slug was placed in a clean 50 ml Falcon tube that was sprayed three times with deionized water using a perfume atomizer to moisten the tubes. The tube was closed halfway with a lid to facilitate airflow. Each tube contained one slug. For control and MeJA treatments, tubes were prepared in a similar manner but without slugs. After 20–22 h, the slug was removed from each tube. The locomotion mucus and water were scraped to the bottom of the tube using a stainless-steel spatula. After that, 10 ml of deionized water and two glass beads were added, and the tubes were capped. The tubes were placed on a vortex (VWR International LLC) and vigorously shaken for 10 min. Similar procedure was followed for control and MeJA treatments. The locomotion mucus–water solution and water solution were used right away to treat the seedlings. Great care was taken not to apply MeJA in the vicinity of seedlings assigned to the other two treatments. On the day of cue application, seedlings allocated to MeJA treatment were placed on one end of the hallway, and seedlings assigned to control and slug mucus treatments on the other end of the hallway, separated by a door. Depending on the treatment to which seedlings were assigned, they received one of the three following cues:

1. Control seedlings—carrier in water as foliar spray + 10 ml of water solution as soil drench
2. MeJA seedlings—5 mM MeJA and carrier in water as foliar spray + 10 ml of water solution as soil drench
3. Slug mucus seedlings—carrier in water as foliar spray + 10 ml of locomotion mucus–water solution as soil drench. (Note: a different slug was used to generate the cue each time). Slug mucus was applied to seedlings as a soil drench to mimic the natural field settings, whereby slugs, as they move through the forest, leave a trail of mucus which subsequently seeps into the soil after rain, irrigation, etc.

After 4 h of cue application, seedlings were moved back into the greenhouse. Cues were applied every 3 days in the morning for a total of five applications (Supplementary Fig. S1). Seedlings were watered every day, except on days when cues were applied.

### Growth and total phenolics

#### Growth

Relative height, root–collar diameter, leaf area, aboveground- and belowground-biomass were measured at four different timepoints: 3, 16, 30, and 88 days post final cue application. Three to four replicates were used for the first three timepoints, and the last timepoint used six replicates. At each timepoint, a subset of seedlings from each treatment was randomly selected, height of individual seedling was measured, and then each seedling was harvested by tissue type (leaves, stem, and roots). All leaves of each seedling were photographed, pooled, and placed in individual coin envelopes. Roots were washed with tap water to remove soil. All the harvested seedling parts were placed on ice in a cooler and transported to the lab.

For each seedling the following growth parameters were measured: relative height, root–collar diameter, leaf area, aboveground biomass, and belowground biomass. Relative height calculated as height of an individual seedling at sampling (timepoint)—initial height. Root-collar diameter was measured with a caliper to the nearest 0.1 mm. The total leaf area of each seedling was measured using ImageJ software. For each seedling, the stem was separated from the roots, and fresh masses of leaves, stems, and roots were recorded using an analytical balance with 0.1 mg precision (Mettler Toledo, OH, USA). Aboveground biomass was calculated by combining leaf and stem mass.

On the days growth was measured, leaf mass was taken first, then the first pair of leaves (>75% of fully expanded) from the apex was separated, and one leaf in the pair was used for bioassays with *L. dispar* on the same day. The other leaf was placed in a coin envelope and vacuum-dried in a lyophilizer for approximately 24 h. Following, envelopes containing leaves were placed in Ziploc bags, stored in −20°C freezer, and processed to determine total phenolic concentrations.

#### Total phenolics

Vacuum-dried leaves were ground to a fine powder with a ball mill grinder (Retsch MM 400) at 25 hz for 1 min. Leaf sample 10 mg (± 0.5 mg) was transferred to a 1.5 ml microcentrifuge tube and 1 ml of 80% methanol was added. Then, samples were vortexed for 15 s, sonicated for 30 min in ice-cold water, and centrifuged at 10 000 rpm for 5 min. To measure total phenolics, 20 µl of leaf sample was added to a test tube, followed by 1 ml of deionized water and 500 µl of Folin-Ciocalteu reagent. The sample was vortexed for 15 s and placed in the dark for 5–8 min. Following, 1.5 ml of 20% sodium carbonate was added. The final total volume was made to 10 ml by adding water. The samples were vortexed again for 20 s and left in the dark for 1.5 h. All samples were done in triplicates. Finally, 200 µl of each sample was pipetted into a 96-well plate, and the absorbance was read at 760 nm using Spectramax M3 Plate Reader (Molecular Devices LLC, CA, USA). A gallic acid standard curve (*y* = 0.116*x* + 0.002, *R*^2^ = 0.998) with different concentrations of gallic acid (0–6 mg/ml) was developed and used to calculate the total phenolic concentration, which is expressed as mg gallic acid equivalents per g dry leaf mass. All chemicals were purchased from Sigma-Aldrich, MO, USA, except sodium carbonate, which was purchased from Fisher Scientific, NJ, USA.

### Herbivory no-choice assays

Spongy moth (*L. dispar*) eggs were obtained from Otis Laboratory, USDA-APHIS, MA, USA. After hatching, neonates (<24 h old) were carefully transferred with a fine paint brush to a 30-ml plastic cup (Sweetheart Cup Company Inc., MD, USA) that contained artificial diet made of wheat germ and casein (Bio-Serv, Frenchtown, NJ, USA). Three larvae were reared per cup. To synchronize the larval stage, third instars (within 12 h from molting) were used for the no-choice assays. On the day of bioassay, one larva from each cup was removed, housed individually in a clean plastic cup, and starved for 4 h. For each replicate, three leaf discs excluding midrib were cut using a cork borer (size 14), and then the leaf discs were placed on a 90 mm Petri dish that was lined with moistened filter paper. After the starvation period, each larva was weighed and placed in a Petri dish that contained leaf discs from one of the three treatments, and the Petri dish was closed with a lid and sealed with parafilm. Larvae were left to feed for 16 h and then removed from the Petri dish, starved for 4 h, and their mass was recorded. For timepoints, 3, 16, and 30 days post final cue application, three or four replicates and for timepoint 88 days post final cue application, four or five replicates per treatment were performed. The relative larval growth rate was calculated following the Lariviere *et al.* (2015), where the relative growth rate was the difference between natural-log-transformed final mass and natural-log-transformed initial mass, divided by the number of days of bioassay.

### Statistics

Each seedling-growth trait (relative height, root–collar diameter, leaf area, aboveground biomass, and belowground biomass) and total phenolics were analysed separately using linear models with cue type, timepoint, and their interaction as main effects. Statistical significance was determined using Anova function in ‘car’ package (Fox and Weisberg 2019). Linearity of the residuals was checked, and data on seedling traits except leaf area and total phenolics were log-transformed. Multiple mean comparisons were made using least square means (Russell 2016). For *L. dispar* relative growth rate, we ran separate analyses at each timepoint following one-way ANOVA, since we used a new cohort of plants and insects each time. All residuals were evaluated for normality and homoscedasticity using Shapiro–Wilks test and Levene’s test, respectively. In addition, linearity of residuals using Q–Q plots was checked. All data analyses and figure production (packages: ‘ggplot2’ & ‘cowplot’ (Wickham 2016, Wilke 2024)) were performed using R Statistical Software (v 2023.12.1; R Core Team 2023).

## Results

### Growth and total phenolics

#### Growth

**Figure 1:**
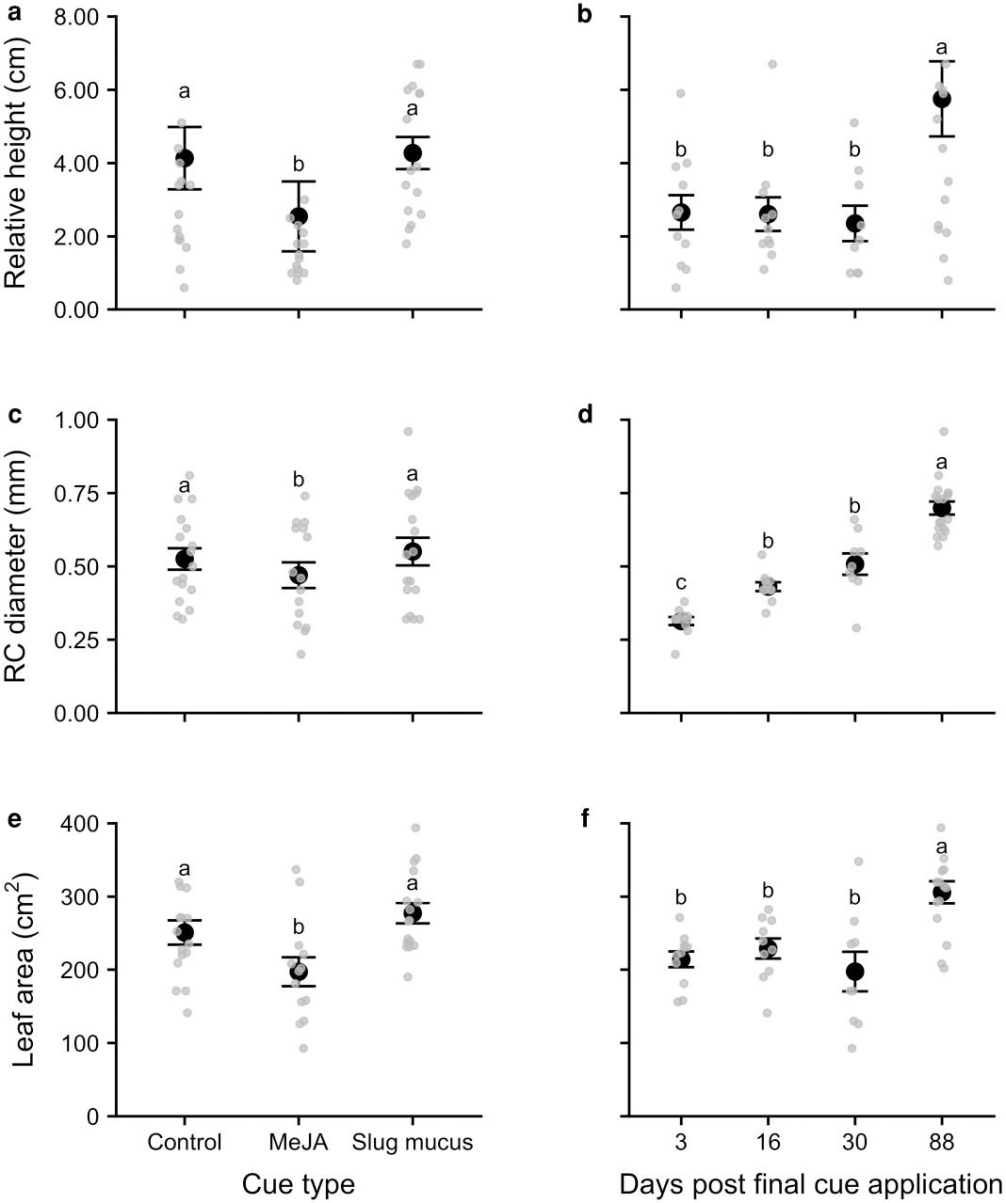
Mean (± SE) (a, b) relative height, (c, d) root–collar diameter (RC diameter), and (e, f) leaf area of seedlings either treated with 5 mM methyl jasmonate (MeJA), locomotion mucus of the dusky slug, *Arion subfuscus* (slug mucus) or left untreated (control) at four different timepoints (3, 16, 30, and 88 days post final cue application) in sugar maple, *Acer saccharum*. In each panel, different lowercase letters indicate significant differences among the treatment groups (*P* < 0.05). Single data points are indicated by solid circles in grey. For each cue type, at 3, 16, and 30 days post final cue application and at 88 days post final cue application *n* = 3 or 4 and *n* = 6, respectively.

Both cue type and timepoint but not their interaction had a significant impact on several seedling-growth traits: relative height, root–collar diameter, and leaf area (Table 1). Specifically, seedlings treated with MeJA had 38% less relative height, 11% smaller root–collar diameter, and 21% less leaf area compared to control seedlings (Fig. 1a, c and e; Supplementary Table S1). In contrast, no significant differences were detected between slug mucus-treated seedlings and control seedlings (Fig. 1a, c and e; Supplementary Table S1). Over the course of the experiment, all seedlings increased in relative height, root–collar diameter, and leaf area (Fig. 1b, d and f; Supplementary Table S1).

**Table 1:** Results of linear effects model of cue type (untreated control, 5 mM methyl jasmonate or locomotion mucus of the dusky slug, *Arion subfuscus*) at four different timepoints (3, 16, 30, and 88 days post final cue application) on seedling traits: relative height, root–collar diameter, leaf area, aboveground biomass, belowground biomass and total phenolics of seedlings in sugar maple, *Acer saccharum*.

Seedling-growth trait	Effect	*df*	*F*	*P*
Relative height	Cue type	2	8.39	**0**.**001**
	Timepoint	3	5.66	**0**.**003**
	Cue type × Timepoint	6	0.50	0.806
Root-collar diameter	Cue type	2	11.04	**0**.**0001**
	Timepoint	3	91.41	**<0**.**001**
	Cue type × Timepoint	6	1.15	0.354
Leaf area	Cue type	2	14.35	**<0.001**
	Timepoint	3	17.16	**<0.001**
	Cue type × Timepoint	6	1.52	0.203
Aboveground biomass	Cue type	2	17.16	**<0.001**
	Timepoint	3	56.35	**<0.001**
	Cue type × Timepoint	6	3.71	**0.005**
Belowground biomass	Cue type	2	8.69	**0.001**
	Timepoint	3	240.61	**<0.001**
	Cue type × Timepoint	6	3.00	**0.017**
Total phenolics	Cue type	2	15.32	**<0.001**
	Timepoint	3	4.96	**0.006**
	Cue type × Timepoint	6	2.68	**0.003**

Significant *P* values (<0.05) are highlighted in bold.

**Figure 2:**
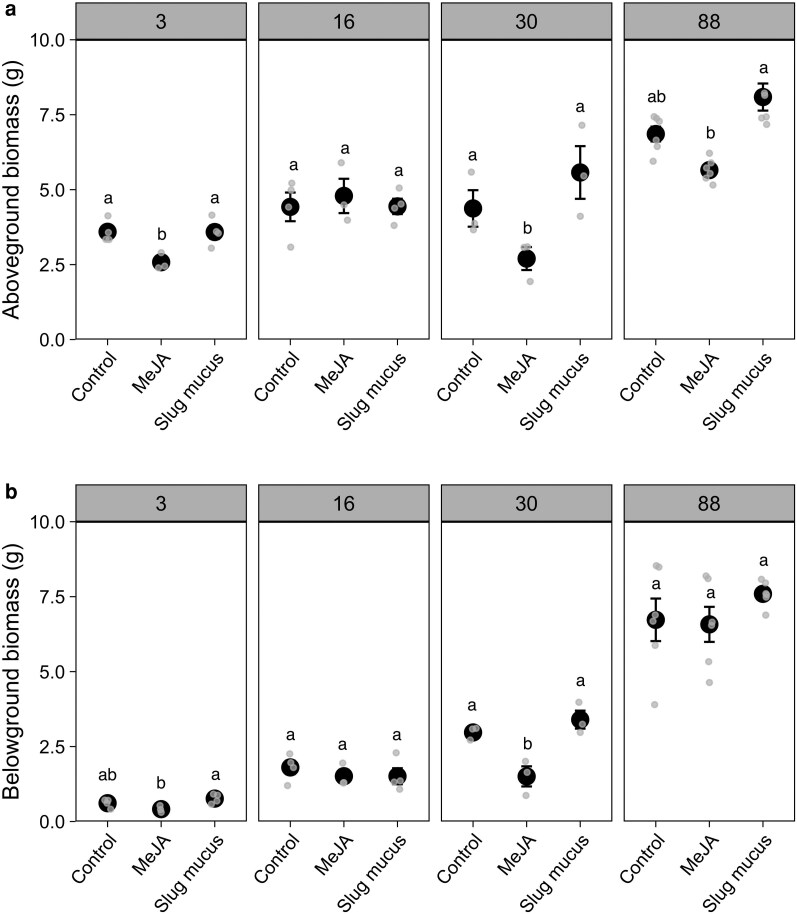
Mean (±SE) (a) aboveground biomass and (b) belowground biomass of seedlings either treated with 5 mM methyl jasmonate (MeJA), locomotion mucus of the dusky slug, *Arion subfuscus* (slug mucus) or left untreated (control) at four different timepoints (3, 16, 30, and 88 days post final cue application) in sugar maple, *Acer saccharum*. In each panel, different lowercase letters indicate significant differences between treatments (*P* < 0.05). Single data points are indicated by solid circles in grey. For each cue type, at 3, 16, and 30 days post final cue application and at 88 days post final cue application *n* = 3 or 4 and *n* = 6, respectively.

For aboveground and belowground biomass, there was a significant effect of cue type, timepoint, and cue type by timepoint interaction (Table 1, Fig. 2). At day 88, seedlings treated with MeJA had on average 18% lower aboveground biomass relative to untreated seedlings, which means that they did not recover from MeJA treatment (Fig. 2a). For belowground biomass, MeJA generally inhibited root growth. At 3 and 30 days post final cue application, belowground biomass was significantly lower in MeJA treatment compared to control and slug mucus treatments, but at 88 days post final cue application the belowground biomass did not differ among treatments (Fig. 2b). In contrast, the belowground biomass of slug mucus-treated seedlings and untreated seedlings did not differ significantly at any timepoint.

#### Total phenolics

**Figure 3:**
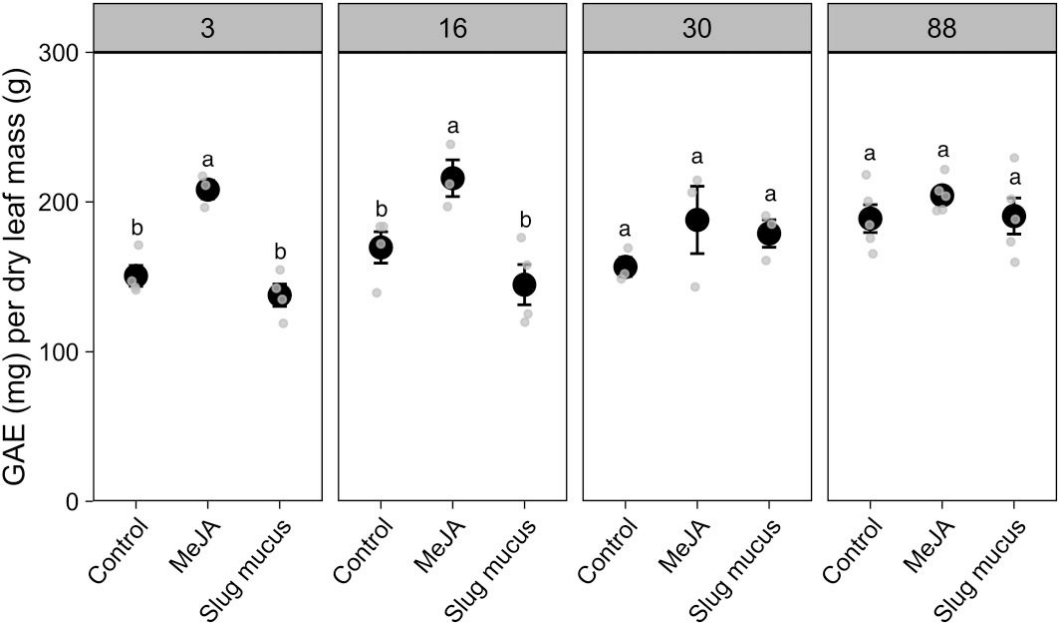
Mean (±SE) concentration of total phenolics expressed as gallic acid equivalents (GAE, mg) per g of dry leaf mass in the seedlings either treated with 5 mM methyl jasmonate (MeJA), locomotion mucus of the dusky slug, *Arion subfuscus* (slug mucus) or left untreated (control) at four different timepoints (3, 16, 30, and 88 days post final cue application) in sugar maple, *Acer saccharum*. Different lowercase letters indicate treatment means differ significantly (*P* < 0.05). Single data points are indicated by solid circles in grey. For each cue type, at 3, 16, and 30 days post final cue application and at 88 days post final cue application *n* = 3 or 4 and *n* = 6, respectively.

The concentration of total phenolics in leaves was significantly affected by cue type, timepoint, and the interaction between them (Table 1). At 3 and 16 days post final cue application, concentration of total phenolics was significantly higher in seedlings treated with MeJA relative to others (Fig. 3). But these differences dissipated by days 30 and 88. However, the concentration of total phenolics did not differ significantly between slug mucus-treated seedlings and untreated control seedlings at any timepoint.

### Herbivory no-choice assays

**Figure 4:**
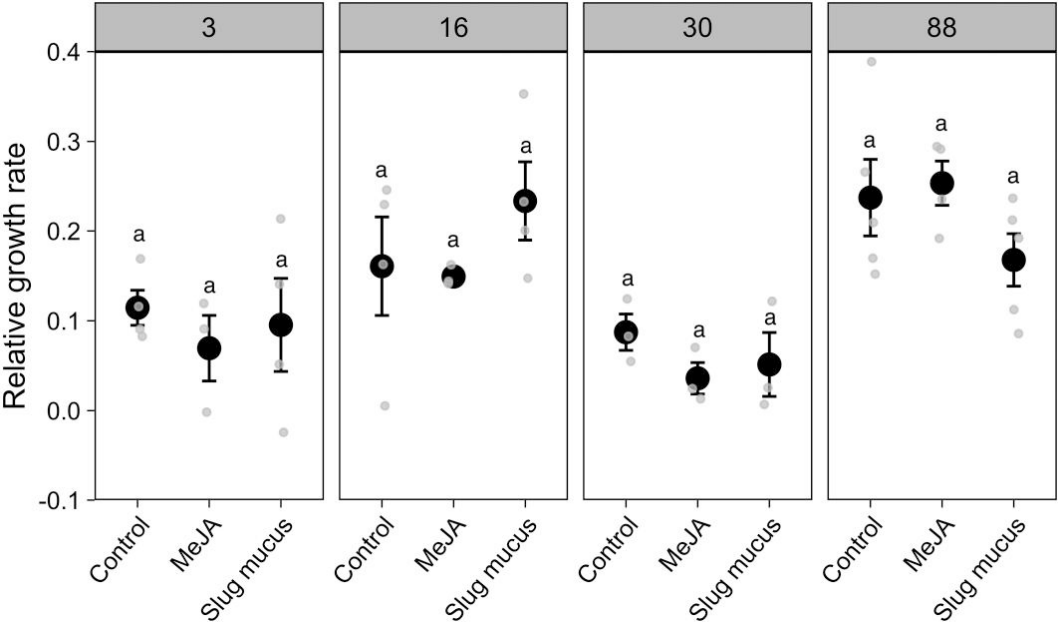
Relative growth rate of the spongy moth, *Lymantria dispar* larvae fed on leaves taken from seedlings either treated with 5 mM methyl jasmonate (MeJA), locomotion mucus of the dusky slug, *Arion subfuscus* (slug mucus) or left untreated (control) at four different timepoints: 3, 16, 30, and 88 days post final cue application in sugar maple, *Acer saccharum*. Same lowercase letters indicate treatment means did not differ significantly (*P* > 0.05). Single data points are indicated by solid circles in grey. For each cue type, at 3, 16, and 30 days post final cue application and at 88 days post final cue application *n* = 3 or 4 and *n* = 4 or 5, respectively.

Relative growth rate of *L. dispar* larvae did not differ among treatments at any timepoint (ANOVA: timepoint 3: *F*_2,8_ = 0.31, *P* = 0.742; timepoint 16: *F*_2,8_ = 1.05, *P* = 0.393; timepoint 30: *F*_2,6_ = 1.05, *P* = 0.406; and timepoint 88: *F*_2,11_ = 1.76, *P* = 0.218; Fig. 4).

## Discussion

Although induced defences are widespread in plants (Karban 2020) and plants use a variety of cues to initiate the onset of defence (de Bobadilla *et al.* 2022, Karban 2020), the responses of deciduous broad-leaved woody plants, especially in seedlings, are still relatively unexplored. Indeed, we provide evidence that young *A. saccharum* seedlings are inducible but depend upon the type of cue. Specifically, *A. saccharum* seedlings treated with MeJA had higher levels of total phenolics in their leaves, and seedling growth decreased at 3 days post cue application. Although MeJA application led to an increase in total phenolic concentrations during the first and second sampling periods, slug mucus did not cause changes in total phenolics at any timepoint. Moreover, neither MeJA nor slug mucus treatments decreased *L. dispar* caterpillar growth rate.

As expected, MeJA caused changes in seedling growth and total phenolics. The effects of MeJA on induced resistance at different plant developmental stages have been extensively studied in herbaceous plant species (see Giri and Zaheer 2016, Rani and Murali-Baskaran 2025). Moreover, there is evidence that seeds respond to plant elicitors such as MeJA, which can lead to the induction of chemical defence responses and resistance against herbivores in herbaceous plants (Erazo-Garcia *et al.* 2021, Ray *et al.* 2025, Strapasson *et al.* 2014). Such induced resistance, however, lasted for a short period and disappeared over time with plant development (Kraus and Stout 2019, Paudel *et al.* 2014). Studies on conifers and deciduous broad-leaved woody plants that were mostly conducted on older trees also show that MeJA treatment reduces plant growth and increases plant defence expression (Gould *et al.* 2009, Heijari *et al.* 2005, Jiang and Yan 2018, Martin *et al.* 2002, Moreira *et al.* 2009, Yin *et al.* 2016), consistent with the results of this study related to MeJA-induced growth and defence responses in sugar maple seedlings. While *A. saccharum* seedlings responded to herbivore cues through changes in some growth traits and total phenolics, these induced responses varied depending on the herbivore cue.

Induced responses seen in *A. saccharum* seedlings were transient and decayed over time. For example, by 30 days post final cue application, levels of total phenolics in MeJA-treated seedlings did not differ significantly from other treatments. Induction of chemical defences immediately following herbivore cue perception may be a strategy to reduce the risk of herbivory attack or damage caused by herbivores in *A. saccharum* seedlings as evidenced in other plant species (Calf *et al.* 2020, Orrock *et al.* 2018, Pellegrini *et al.* 2024). A subsequent decay is expected if the threat of herbivory passes and there is no actual herbivore damage. Moreover, MeJA-treated seedlings had lower aboveground- and belowground-growth at 3 days post cue application. Such short-term negative impacts of exogenously applied MeJA on plant growth have been well-documented in different plant species (Heijari *et al.* 2005, Rani and Murali-Baskaran 2025 for review). Over time, these responses may disappear depending on the plant species, timing of application, and plant traits measured (Feng *et al.* 2012, Worrall *et al.* 2012).

MeJA not only immediately slowed growth of seedlings, but MeJA application led to differences in biomass that were still evident 88 days after cue exposure. Interestingly, these changes in biomass allocation depended upon which part of the plant was considered: belowground biomass was initially (at 3 days post final cue application) lower but was similar by the end of the experiment (at 88 days post final cue application), whereas aboveground biomass except at 16 days post final cue application was lower. In plants, shifts in resource investment to above- and belowground plant parts in response to herbivory have been well-documented (Gómez *et al.* 2012, Oba *et al.* 2006, Pastore and Russell 2012, Thomas *et al.* 2017). Perhaps the greater allocation of resources to belowground parts over aboveground parts, as seen in MeJA-treated seedlings, is a strategy to sequester resources away from the site of attack (Korpita *et al.* 2014, Orians *et al.* 2011). We note that the time of year may have also affected regrowth potential. The lack of difference in belowground biomass between MeJA treatment and control treatment by 88 days post final cue application could be explained because later in the season temperate woody plants invest less in new leaves and generally prioritize storage in roots, which could enhance overwinter survival and/or growth the following year (Furze *et al.* 2019, Loescher *et al.* 1990, Thomas *et al.* 2017, Tixier *et al.* 2019). Further research would be valuable to distinguish between these factors.

It is important to understand how contrasting cues impact growth and defence in deciduous broad-leaved woody seedlings as induced responses in growth and defences vary with herbivory species. In contrast to MeJA, slug mucus did not negatively affect seedling growth or total phenolic levels as we had hypothesized. These results contrast with previous work exploring the response of herbaceous plants to molluscs. For example, undamaged *B. nigra* and *S. lycopersicum* plants exposed to snail, *Helix aspersa*, locomotion mucus exhibited reduced growth and higher defence expression (Orrock 2013, Orrock *et al.* 2018). Pellegrini *et al.* (2024) also found that *A. subfuscus* mucus had a negative effect on seedling biomass in *B. nigra*. Given that *Arion lusitanicus* mucus can activate JA defences when applied to *A. thaliana* leaves (Falk *et al.* 2014), the increased resistance of *B. nigra* may have been due to an upregulation of defence. Clearly, slug mucus can activate plant defence responses in herbaceous plants, but in this study, we found no evidence that slug mucus leads to induced defences in sugar maple seedlings. Consistent with these results, in another study, we found that locomotion mucus does not impact seed germination or the growth of even younger maple cotyledons (Headrick *et al.* 2026).

There are several non-mutually exclusive explanations for this lack of response of *A. saccharum* seedlings to slug mucus. First, there could be strong selection for early production of constitutive defences in long-lived shade-tolerant species if tissue loss has a strong negative effect on survival (Dudt and Shure 1994, Imaji and Seiwa 2010). In fact, this may explain the constitutively high levels of phenolics in sugar maple (Barbehenn *et al.* 2009), yet induction is possible, as evidenced from the MeJA-treated seedlings. Perhaps the combination of high phenolics (Fig. 3) and the rapid lignification of leaves (Calf *et al.* 2020, Peeters 2002) deters slugs and makes induced responses less beneficial. Further work on the ontogeny of defence in sugar maple seedlings and other shade-tolerant woody species is needed. Second, although slugs are one of the important causes of seedling mortality of woody plants in temperate forests (Albrectsen *et al.* 2007, Gardescu 2003, Pigot and Leather 2008), the window of vulnerability of seedlings appears to be narrow. Previous studies have shown that slugs prefer young seedlings over older seedlings and mature plants (Albrectsen et al. 2004, Elger et al. 2009, Orians et al. 2010). Albrectsen *et al.* (2004), for example, found that larger vigorously growing willow seedlings had higher phenolics and were avoided by slugs. Since we have only observed slugs feeding on sugar maple cotyledons (Headrick, K. and Pellegrini, B., personal observation), there may be little reason for larger seedlings to respond to slug mucus. Third, it is possible that all woody plant species may be less responsive to slug mucus. Although our approach is capable to altering growth and defence of herbaceous plants (Pellegrini *et al.* 2024), higher exposure (i.e. locomotion mucus collected from more number of slugs, e.g.) may be required to induce woody plants. This is evident from the fact that higher concentrations of MeJA are often used when treating woody plants compared to herbaceous plants (e.g. Cai *et al.* 2014, Chen *et al.* 2021, Gómez *et al.* 2010, Moreira *et al.* 2009, Rowe *et al.* 2012). Moreover, Nosko and Embury (2018) reported that chemical induction of total phenolics occurred only when loss of plant biomass exceeded a threshold. Finally, it is possible that diet might influence the locomotion mucus chemistry, and in turn induced responses. Although we used lettuce-fed slugs, rather than maple-fed slugs, to avoid the possibility of seedlings responding to maple ‘damage’ cues, this possibility warrants future research.

The lack of effect of these herbivore cues on *L. dispar* third instar performance was unexpected. This may reflect higher tolerance of older larvae to changes in host quality (Barbehenn *et al.* 2013). Roth *et al.* (1994) studied *L. dispar* performance across woody plants and found that the relative larval growth rates of the fourth instars did not differ among treatments even when total phenolic concentrations differed. This may also reflect the fact that among different hardwood species, sugar maple is a less preferred host of *L. dispar* (Hamilton and Lechowicz 1991, Hough and Pimentel 1978, Liebhold 1995, Shields *et al.* 2003). Finally, differences in leaf development may have contributed to high variability in performance across the timepoints. For this no-choice study, the topmost leaf that was at least 75% expanded was used. As a result, some leaves were fully expanded and dark green, while others were still expanding, light green, and succulent. Thus, differences in nutrient content, phenolics, and lignification may have contributed to differential performance among timepoints.

In summary, our results highlight the differences in induced responses between MeJA and slug locomotion mucus treatments. While non-damage herbivore presence cues can be important cues in some plant systems, these cues appear to be trivial to sugar maples despite the fact that slugs are important herbivores of young seedlings. Further research needs to be conducted to elucidate the mechanisms involved in the perception of herbivory in *A. saccharum*.

## Supplementary Material

plag026_Supplementary_Data

## Data Availability

Raw dataset and the R code used are available on Figshare: https://doi.org/10.6084/m9.figshare.31831063
