# Supplementary material for: Effects of contrasting herbivore cues on seedlings of a long-lived woody tree: growth, chemistry, and resistance to herbivores
Source: AoB Plants. 2026 Jun 10;18(3):plag026. doi: 10.1093/aobpla/plag026 (PMC13278774; doi:10.1093/aobpla/plag026)
Supplement: plag026_Supplementary_Data [file plag026_supplementary_data.pdf]

Table S1. Mean (± SE) relative height (cm), root-collar-diameter (RC diameter, mm), and leaf area (cm^2) of seedlings either treated with 5mM methyl jasmonate (MeJA), locomotion mucus of the dusky slug, *Arion subfuscus* (slug mucus) or left untreated (control) at four different timepoints (3, 16, 30, and 88 days post final cue application) in sugar maple, *Acer saccharum*.

Seedling-Growth-Trait	Cue type			Time point			
	Control	MeJA	Slug mucus	3	16	30	88
Relative height	4.14 ± 0.85	2.55 ± 0.95	4.28 ± 0.44	2.65 ± 0.47	2.61 ± 0.46	2.36 ± 0.48	5.75 ± 1.02
RC–diameter	0.53 ± 0.04	0.47 ± 0.04	0.55 ± 0.05	0.31 ± 0.01	0.43 ± 0.02	0.51 ± 0.04	0.70 ± 0.02
Leaf area	250.63 ± 16.58	197.12 ± 19.72	277.06 ± 13.92	214.09 ± 10.75	228.80 ± 13.72	197.40 ± 26.97	305.73 ± 15.27

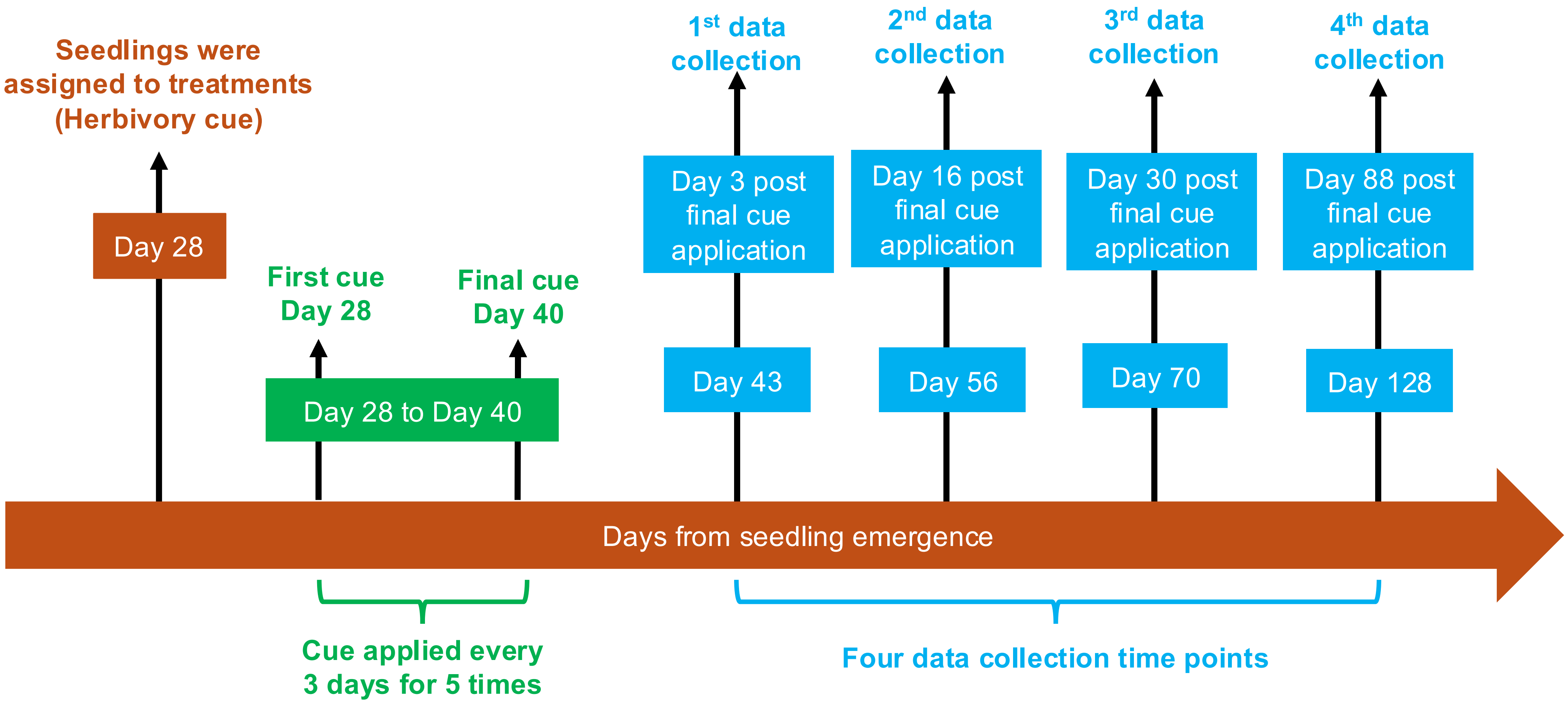

Figure S1. Diagram showing the days of herbivory cue application and data collection timepoints
